# Physiology, Pathology and Therapeutics of Muscular Exercise

**Published:** 1858-08

**Authors:** W. H. Byford


THE CHICAGO

MEDICAL JOURNAL.

VOL. I. AUGUST, 1858. No. 8.

### ORIGINAL COMMUNICATIONS.

### ARTICLE I.

### PHYSIOLOGY, PATHOLOGY AND THERAPEUTICS OF MUSCULAR EXERCISE.

(Read before Cook Co. Medical Society, and published at their request.)

BY W. H. BYFORD, M. D.

### PHYSIOLOGY.

*Phenomena.*—When an individual exercises actively to the extent of producing perceptible effects, there are a glow of redness on the surface, an increase of temperature, perspiration and respiration. These circumstances are apparent to the observation of the most careless observer. Upon closer scrutiny into the manifestations of muscular exercise, we find the heart and arteries beating rapidly and several of the secretions increased in quantity, and their peculiar products enhanced to a considerable extent. The urinary secretion, the secretion of the liver, skin and the pulmonary excretion, are all carried on more actively than in a state of repose, while the secretion and excretion of the alimentary mucous membrane are diminished. I speak now of an amount of exercise that produces quite obvious phenomena. I am inclined to the opinion, however, that moderate and prolonged exercise increases all the excretions beyond what they are in protracted repose. These are the more obvious phenomena of exercise. The interesting question now arises, how are they produced? What are the intimate circumstances transpiring during the exhibitions mentioned? Voluntary motion of the muscular organs is preceded by innervation, the production and transmission of the power of the will, and the contraction of the muscular fibrilla. Every functional action of whatever kind occur as the effect of cell change. This proposition is true in relation to the production of the power of volition in the brain and the contractions of the muscular fibrilla, as well as the secretory processes. Each time the nervous or mental action is effected, cells receive from the cerebral tissue and impart to it certain elaborate and effete material; the latter derived from the disintegration of the nervous or cerebral mass, and the former to be used for its repair. So also the muscular tissue at each contraction receives through the medium of cell action or change, material from the blood intended for the repair of the damage sustained during contraction, and throws into the circulating fluid through the same agency the effete substance lost in its disintegration. We may state it as a correct proposition, that one of the necessary conditions of action in an organ is organic destruction or disintegration and repair. The disintegration occurring simultaneous to and most likely being the result of action in each particular instance, and the repair a necessary prerequisite to future action, the organ in this way would constantly retain its integrity of structure, notwithstanding ever-recurring change, as the effect of action. Chemical or vital affinities, or both, between the changing tissues and the constituents of the blood, effects this interchange of substances as rapidly as the acting organs are called upon to perform their functions. This principle, it will be seen, precludes the probability of organic change being left behind as the result of action while the organ remains capable of its duties, and teaches the doctrine that so soon as the play of affinities from any cause is so weakened, or rendered impracticable from want of nervous stimulus or blood products, that repair is not perfect, the function will be imperfectly discharged, or entirely cease. Any disease of the nervous system that disturbs its presiding capacities, as in fevers, severe shocks, pressure upon the brain, etc., thus incapacitates a certain portion or the whole of the system, owing to the extent and gravity of the nervous lesion for functional action. The reciprocal affinities in this case are lost for want of nervous agency. If the nervous system be but slightly affected, the affinities are merely more tardy; if gravely, they are entirely suspended. Thus, the blood and muscle may both possess all of their own special chemical and vital properties, and yet not be capable of acting and reacting upon each other, then the muscular contraction must cease.

You will recognize in this statement of conditions the pathology of paralysis from cerebral congestion, pressure from effusion, or pressure or inflammation on or in a large nervous trunk. Another source of functional tardiness and even suppression, is the want of the proper material to sustain the organs in a state of integrity under functional action. If the blood is prevented from elaborating the various materials which go to form nerve and muscle, from the presence of some poison as carbon, urea, bile, etc., the functions are performed imperfectly, and if carried to a great degree of contamination, the blood is entirely incapable of supporting functional action, and these all cease. If also from want of nutrition, the blood does not receive the substances from which muscle, nerve or other organic elements may be produced, these last will not be elaborated, and hence the chemical affinities resulting from their presence could not take place, the muscle cannot act with energy, if at all, the nervous functions are performed irregularly and imperfectly, the secretions are scanty and unhealthy, because the material out of which the repair after functional changes in the organ is not supplied, and all succeeding efforts become more and more imperfect until they cease entirely. This is the condition of things in anemia, and it accounts for the tardiness of the bowels, the imperfect secretion of the liver and other intestinal organs, the whole phenomena in fact of that state of the circulating mass. This play of affinities between the circulating blood in the capillary bloodvessels and the changing tissue, the fibrilla of the muscles and the nervous structures, engaged in muscular exercise, is the starting point, I think, and a sufficient basis upon which we can found a rational and I hope not unprofitable explanation of all the phenomena observed.

In the composition of the blood are contained all of the separate ingredients of all the tissues in the body either elaborated, so as to immediately enter into the composition of these tissues while passing them in the capillary tubes, or to be readily and easily changed by the chemical or vital affinities, or both, of the organs to which they are appropriated. These ingredients must possess distinctive differences to a certain extent at least, so that we may say with great propriety, that in the blood are contained muscular fibre, nerve fibre, bile, urine, bone, and so on to all the products of functional action and nutrition, either ready formed, only awaiting separation, or formed, requiring elaborating mutation by means of cell action, to be appropriated as needed, for the formation or repair of structures. Another proposition forces itself upon our mind in this connection as necessarily true, viz: that these ingredients must exist to a limited extent of quantity only and something of definite proportions to the probable wants of the various organs used, owing to the habits to some extent of the individual. The man, for instance, whose muscle is called into action to a greater extent than any other system of organs, will require more food from which muscle tissue may be formed, and his chyliferous and sanguiferous peculiarities will be that of muscular elaboration, those organs will become large and be kept in a state of perfect nutrition, at the expense perhaps of the brain. Individuals, on the other hand, whose functions are mental exclusively, will have an abundance of neurine in the blood ready for use, while the muscle cannot be nourished to such an extent as to admit of more than very moderate muscular exercise. We often have examples of partial impoverishment of the blood of this kind brought about by habits. If the man of muscle was called upon to exercise his mind long and intensely, he would soon have the headache, lassitude and exhaustion, in fact as readily become exhausted with this kind of unaccustomed effort as the brain worker would if required to labor actively at anything that required muscular exertion. These habits and capacities may be at least partially changed by gradually bringing about different habits in the two individuals, and with it different sorts of nutrition and the necessity of different diet. It must be remembered that all these ingredients, intended for different purposes, are thoroughly mixed together and circulate in company to all parts of the system, so that through the capillary vessels of the brain we have the neurine with the muscular, bilious and other materials, instead of the neurine alone. The affinity of the nerve fibre for its peculiar material of support, through the agency of cell metamorphoses, takes up from the blood its own nutritive elements and leaves the other ingredients to pass along and be elsewhere appropriated for other purposes. Of course it is supposable that only just so much material is abstracted as is needed for repair and growth and no more, and if the blood is still rich in such ingredients, much of them pass on to be returned in the general course of the circulation. In like manner the material for the formation of muscle is arrested and appropriated as needed, as with every other peculiar material. The more actively the function of any organ is proceeding, the more rapid is the play of affinities between its intimate structure and the blood in the capillaries. The blood, by means of the attraction of the acting organ in its atomic changes, is attracted into the capillaries in increased quantities, the attraction being always from the arterial extremities of these minute tubes. It is thus drawn from the arteries by this attractive force. So soon as the chemical or vital changes have resulted in the repair of the acting structure, this artero-tissual attraction ceases, but now there is thrown into the stream of passing blood the effete material resulting from tissual disintegration, where there is another force developed, a kind of tissue-venous repulsion which urges it forward in the veins. These are the main elements of force in the capillary circulation, the attraction of interstitial and separative nutrition and the repulsion of interstitial disintegration and distinction. Now to justly estimate the effect of these forces, it will only be necessary to recollect that the tubal area of the capillary vessels, compared to that of the arteries whence they are supplied with blood, is four hundred times larger. Hence, if nutritional attraction is feeble—which it certainly is—when exercised by a single cell, it becomes enormous when billions of them combine in pulling in the same direction. In fact, I have sometimes fancied that if all the organs could act simultaneously through all their capillary vessels, so much blood would be thrown through the veins upon the heart as to burst it from excessive accumulation. If, on the other hand, all nutritive attraction were to cease, the blood would accumulate in capillaries, cease to evolve caloric and the circulation to stop. Indeed, it is quite probable that such is the condition of things in extensive congestive collections in different parts of the body, or of a general character. In cholera, in which there is general congestion, this seems to some extent to be the case. The blood stops in the capillaries and passes through them with great difficulty, either on account of its altered condition or the arrest of nutritional changes going on necessary to keep up the circulation, the watery portions transude through the skin and mucous membrane, while there is a complete want of response to medicinal appliances, as though the functions of the stomach, both absorbent and digestive, had entirely ceased. In repose, or an accustomed degree and kind of exercise, the capillary flow of blood is such as to keep up a pulse, say to 75 or 80 beats the minute. In walking or standing, in persons accustomed so to do, there is very little acceleration of pulse, but when a larger number of muscles than usual are put into operation, the increased area of capillaries are being acted upon by this nutrition affinity, and consequently a larger amount of blood is poured into the veins and thence into the heart, which is stimulated to greater exertion and also the whole circulation. The relative affinities between the blood and the tissues may be increased therefore by an increase of function in the latter; the more active a muscle is contracting and relaxing, the more rapidly its changes of disintegration and repair is going forward, the greater amount of nutritive material it is attracting from the blood. In persons whose blood is rich in albuminous material for the support of muscle, the less will be the arterial excitement—other things being equal—because the muscles derive their pabulum from a less quantity. If, however, the blood is impoverished in the material necessary for the support of muscle, it is drawn through the capillary vessels more copiously, in order they may receive the same supply. This explains the exciting effect of exercise in anemia. The capillaries must pass a large amount of impoverished blood through the muscles, or a large amount of it is passed through quite rapidly in order that they may derive material for their repair. This being poured into the veins, it goes more rapidly than ordinarily into the heart, which is very excitable, and it is thrown into tumultuous action. There are many good reasons deducible from experiments and observation for believing this to be the true explanation of the physiology of exercise. The mechanical effect of the muscles in pressing the blood forward toward the heart—for on account of the valvular fixtures in the veins passing through the muscles, they cannot press it backward—by squeezing the vein between them, has been supposed to, in some measure, account for the acceleration of the circulation of the blood, and the sweating, heat, etc., to be the effect of the latter phenomenon. But, as I shall show from experiment, this cannot be an explanation in all cases. The secretions and excretions seem to be stimulated to an increase by the mere presence of the ingredients forming their products in a more than ordinary quantity. The kidneys may be stimulated to increased action by a more than ordinary quantity of water in the blood, and by medicinal agents in the circulation. The proper office of the excretory organs, however, is to carry off the waste sustained by functional action in the active organs. They attract the materials of secretion in their capillaries from the arterial extremities of these tubes, and thus may add or produce arterial excitement just as functional action in other organs may do the same. The mere transudation of water through the skin and mucous membranes of the alimentary canal or kidneys as not forming a part of the cell action in secretion, must be considered an exception to this rule. A prominent example of this kind of excitement is the secretion of sugar by the kidneys. Diabetes is always attended by increased arterial excitement, and yet post mortem examinations have not revealed any peculiar inflammatory lesion to account for reaction. In fact, I think we may regard it as a rule, that increase of function above the natural standard of activity involves an accelerated circulation, greater or less according to the circumstances of the case. Exceptions to this rule possibly may be summoned by the memory of some of my friends here to-night, but they do not just now occur to me; and further, the longer functional action continues, the greater will be the velocity of the circulation. This constant increase of circulatory velocity is dependent upon one of the circumstances mentioned above, viz: the definite amount of ingredients intended for the support of functional activity in each organ; something like a definite amount of material for the repair of muscular structure during its acting condition. This substance in a state of preparation is diffused throughout the whole circulating mass. After the process of repair has been kept up during muscular action for some time, the quantity will become lessened, so that the blood to some extent is impoverished of this particular class of ingredients, and a greater amount of blood must be circulated through the muscular fibres in order to impart to them an adequate amount of support,—just as a larger amount of unnutritious aliment is necessary to impart the nourishment that a smaller quantity would afford if more concentrated. This decrease of material and increase of excitement bear a direct proportion to each other, until the blood becomes so much exhausted as to be incapable of supporting the organs under further exertion, when the exercise must cease; not from damaged condition of the acting organ, but from the exhausted condition of the blood. The most interesting proof of this theory of the physiology of muscular exercise is derived from the effects of muscular contraction without motion of the limbs, such general contraction of antagonising muscles as to prevent motion. It will be found by experiment that when an individual is sitting at apparent ease, if he places all the muscles of one lower extremity in a state of great contraction, from the hips to the toes, that his pulse will be accelerated from twenty to twenty-five beats in the minute, and of both lower extremities some ten more. By simply holding one arm horizontally, it will accelerate the pulse from four to ten beats.

I have tried a great variety of experiments of this kind, and I find the larger number of muscles, and the more violent the contractions, the greater the arterial excitement. I have very little doubt but that one half of the soft parts of the whole system in weight is made up of the muscles and their appendages. If this be a true supposition, the area of their capillary vessels is two hundred times larger than the whole of the arteries in the body, and it is not to be wondered at that a small increase in the rapidity of the current in an area of nearly a hundred fold more tabal space—as one leg and hip—should cause perceptibly increased action of the arteries. Another fact of no small importance to the physician deducible from the experiments I have performed and witnessed of this kind is, that this static muscular contraction produced more effect upon the action of the heart and arteries than locomotion. I think this results from the circumstances that the muscles of the legs in locomotion are not all in a state of contraction at the same time, but alternately contract and relax, one set carrying the limb forward while the other set is relaxed. There is yet another remark I desire to make about the physiological action of muscles. Although the body of a muscle may be continued tense for a considerable time, the whole of the fibrilla are not in a state of unvarying rigidity, but the organ, when closely observed, will be seen to tremble and twitch in small portions, each portion being in a different part every instant of time, and upon auscultation the contraction and relaxation of the fibrilla composing its bulk may be heard very plainly. There can therefore be no doubt that a part of the fibrilla are contracting while the others are relaxing and repairing, ready for another contractile effort. For an instant, in a jerk or sudden start no doubt, all the fibrilla may act in unison, and those are the overwhelming contractions that snap asunder the tendo-achilles, or the muscles themselves, or back bones, etc.; but such contraction cannot be maintained even for a moment. Only a part will act at a time on a strain, and the longer the stretch the more ineffectual and feeble this general contraction becomes.

PATHOLOGICAL EFFECTS OF MUSCULAR EXERCISE.

As with every other condition and circumstance which may produce disease, the effects of muscular exercise will vary, and its ultimate morbific action will depend upon many things. Constitutional proportions of the different organs of which the body is composed will modify the effects of exercise, as also the different conditions of them. The tumultuous effects of violent exercise upon persons laboring under pulmonary and cardiac affections would be easily predicted. Dyspeptics, from their incapacity to digest, assimilate and repair the waste resulting from the wear of protracted muscular action, would soon succumb to their exhausting exactions upon nutrition. Persons too whose kidneys, skin, or other emunctories are so damaged as to perform excretion imperfectly, soon poison their own blood to such a degree, under voluntary exertions, that they are soon arrested. Climate has much to do in enabling man to undergo great fatigue without damage. The tropics are peculiarly averse to great and protracted physical labor, probably on account of the air being so rarified as not to oxygenate the blood sufficiently in quantities respirable by an ordinary pair of lungs. Extremely cold climates, although affording oxygen in compact manner, is probably so sedative in its influence as not to be most favorable to enterprise. Experience proves to us what sound physiological knowledge would suggest, that the temperate is much the most favorable zone for man and the enterprises of civilization. All the circumstances necessary to enable man to do more work without damage to his physical system are here combined in that happy manner, as not only thus to capacitate him, but to stimulate him to ambitious and profitable industry. Diet is another one of the most certain modifying circumstances in this respect. Impoverishment, debility, and organic evil is sure to work destruction upon the unfortunate laborer who lives on insufficient fare. Nor can we leave out of the question habit. Laziness incapacitates man for labor, and it would be almost certain to make a lazy man sick to commence hard labor upon any sudden emergency; and we seldom see people physically capable of field or mechanical labor who have led a life of indolence up to mature manhood, and if by mental discipline they force their muscular system into laborious employment, the effects are disastrous in the great majority of instances.

In order, however, to arrive at the proper morbific influence of exercise, we must observe its effects under all the circumstances in which man is placed, while also we bear in mind the degree and kind of exertion.

It will probably be best to commence with a consideration of the acute effects, if I may use the expression, of violent efforts, and afterwards take a view of the more gradual inroads upon health made by protracted toil. Violent muscular contractions often cause tendinous ruptures, as in the case of the tendo-achilles, an instance of which my friend, Dr. C. G. Smith, mentioned to me recently as the result of violent muscular efforts in dancing, as also hernia, etc. Rupture of their own substance: a very interesting case of this accident occurred in the practice of a friend of mine several years ago; the rectus abdominis tore across about the junction of the upper two-thirds with the lower third. Bloodvessels have also been ruptured, and fatal effusion in the brain, chest, or abdomen, the consequence. The heart itself, a powerful muscle, may be lacerated by the distending influx of blood, an instance of which I once knew in the case of an athletic man standing under a heavy load. The patient staggered, fell with his load and never rose. Upon examination, his heart was lacerated and his pericardium distended with extra cardaic blood. Thousands of such disasters are constantly occurring as the accidents of muscular exertion, but it is with the less direct influences that we are now most concerned. It may not be irrelevant, perhaps, to consider for a moment what fatigue is, what its character, and what its import. I mean by fatigue that muscular inconvenience, dependent upon over exertion (local) in the muscles of the legs when it has arisen from walking, in the arms when they have been the members used, etc. This local fatigue is, doubtless, the result of increased vascular action in the parts the capillary vessels have been and continue to be more than ordinarily distended with blood, in order to keep the parts properly supplied with nutritive material, and is caused by the nutritive affinities in the tissues. When this increased vascular action is continued for any length of time, it may become local inflammation and not pass off by rest merely, particularly when other circumstances favor such a state of things. The lungs may also become the focus of local irritation and inflammation as the effect of excitement from exercise. The brain, in fact most of the important viscera, may be thus affected, but I think by far the most morbid effects of exercise are indirectly produced from it. The suppression of excretions, rendered more active by exercise, is a source of aggravated difficulty, if not a primary result of the excitement of exercise. In order to elucidate the idea I wish to convey, I will mention a few facts that are known to exist. During active exercise the blood is loaded with excrementitious substances, as carbon, urea, or its proximate principles, etc., and these are finding outlets through the exhalations from the lungs, skin and increased urinary discharge. The dupurating process is commensurate with the necessities for it, and the blood is maintained in such a condition as is necessary for the support of the vital and nutritive processes of the system to a healthy degree, each emunctory having its own peculiar effete substance to eliminate, and when not interrupted, all goes on well; but should one or more of these emunctories fail to perform their duties, some of the excrementitious substances fail to find their way out of the bloodvessels, and being retained in the mass of blood, to a certain extent poison that fluid, and render it incapable of performing all its complicated offices. This is the reason why suppression of cutaneous excretion proves so disastrous, when the individual is laboring under the wearing effects of violent exercise. We know from experience that it is not simply the stoppage of perspiration, for if perspiration induced by heat be stopped, the effects are not nearly so disastrous as if produced by violent exercise. The recrementitious contents of the blood are smaller in quantity and perhaps less poisonous in character. It should be remembered, however, that there is no time, even in the most calm condition of the functions, but what excrementitious substances do exist in the blood, to an extent of quantity sufficient to prove deleterious to the organism if detained by suppressed excretion. I think it is thus our most violent inflammatory diseases are produced. I desire to be understood as not denying the repulsive effect of suppression of excretion. This is often effective, I have no doubt, in the production of local determination, but I do wish to make a distinction between this effect and the retention of morbid excretion. I wish also to say that much stress is due to the time—in relation to exercise—when this suppression occurs, as to the gravity and direction of the effect produced.

Its chronic effects upon the muscles are well known. It almost invariably results in hypertrophy. The muscles concerned become larger and capable of more than usual power. And no doubt but that nutritional increase may be carried to a great extent if everything else favors. The most interesting, however, is the chronic effects upon the blood, and through it upon the organization at large, or some particular organ or group. This subject has not commanded the attention which ought really to attach to it, and hence there is not much known as to what the effects of excessive muscular exercise, continued for a long time, would be. The instances which usually fall under our observation are such as are combined with privation and often other deleterious influences, so that we cannot separate the evil of the various causes. Excesses in this way, however, must lead to changes in the composition of the blood, abstracting, on the one hand, the material for their support, and thus, in one sense, impoverishing it; and on the other, pouring into the blood excrementitious substances evolved by muscular detritus, and thus injuring its composition. There can be no doubt that exercise for days, without the proper intervals of rest in this way, very much impairs the quality of the circulating mass, and produces morbid determinations, often to the brain, lungs, abdominal viscera, etc. I think there are many reasons for believing that the blood cannot elaborate the materials of digestion poured into it through the stream of chyle, derived through the chylopoietic organs, without some intervals of rest; that the several hours rest of night afford the blood time to recover from the exhausting influence of the exercises of the day; that sleep is a blood repairing process instead of an organ repairing interval from labor. The first effects then of protracted labor would be impoverishment of the blood of certain substances intended for the support of the laboring organs, an increase of the velocity of the circulation, in order to compensate in quantity of blood for the want of richness, until it amounts to morbid excitement, and with local determinations to the organ operating. This may proceed so far as to induce inflammations of the muscles of the brain, etc. The great exertion necessary on the part of the central organ of circulation will induce hypertrophy, with all its evil effects upon the lungs, etc. In a more moderate degree, exercise, to too great an extent compatible with health, would cause simply functional derangements, from the changes of the blood I have just mentioned. The abstraction of certain elements from this fluid must prevent its chemical and vital reactions from taking place normally, in such a way as to keep up any of the functions correctly. I cannot forbear, although it may be not strictly in place, to consider, in this relation, the effects of want of exercise in a short way. The immediate or early effects of inactivity, as observed by the medical man, is in most instances plethora and often local fulness, particularly of the head, scanty secretions and excretions, costiveness, loss of appetite, etc. This condition of things is often followed by depraved secretions, the tongue is coated, various nervous symptoms show themselves in general *malaise.* There is always felt a strong desire to move about, the muscles must be stretched, with yawning and gaping. Now the symptoms of disease that occur as the effect of want of exercise, is very different from those, the result of excesses, in this respect. Excrementitious materials are not retained in the blood, hematoxy does not take place, but there is too great a production of nutritive materials for the organic expenditure, and if digestion remain good and hematization is efficient, more albumen is elaborated than the muscles and nervous system consumes, a larger amount of corpuscles, white and red, exist, than is necessary for oxydation and repair. There is too rich a condition of blood for the meagre demands. The acute effects of this real plethora is to embarrass most of the functions, produce hemorrhages, apoplexy, epistaxis, hemorrhoids, etc. The sudden transition of persons in good health, from an active mode of living to that of sedentary habits, very often lead to diseases of a plethoric character, highly dangerous and often fatal. I will digress here long enough to say that we should always discriminate in local disease, between those arising from suppressed secretion, and consequently in systems possessed of poisoned blood, and those coming up in case where plethora exists, from acute plethora developed in recently assumed habits of inactivity. I think the illustrious General Taylor is an example of the fatal influence of such a change of habits. Doubtless, such instances of disease would bear depletion far better than those originating in other causes.

But supposing that these acute effects are not developed, and the habit of inactivity and its consequences continue, what are we to expect? Now, I consider this one of the most interesting points of pathology, and I regret that the state of our knowledge and my own deficiency of information on this point, prevent me from doing justice to it.

I shall, however, say, in a very brief manner, such things as my experience and reflections enable me. If the opinion I have expressed above be true, that the blood is the great laboratory in which the nutritive materials are prepared ready for their immediate uses, and that each one of these ingredients are prepared in a definite quantity and of separate quality, as for instance such as are intended to repair the muscles, such as go to supply the wear upon the nervous system, etc., and that in order to be readily excreted they must be used for such purposes and cast off as refuse matter, it must follow that these materials will vary with the draw upon them by the acting organs.

I do not assume that ingesta may not be excreted without first passing through these respective structures, but that it is at least unnatural and unusual, and doubtless often gives rise to embarrassment in the secretory and excretory apparatus through which they pass. I believe, too, that many of these different ingredients, after being thoroughly prepared for use, are retained, and raise the albuminous compounds to a higher relative proportion than normal. This, at first sight, would appear of little consequence, but upon closer examination I am induced to believe that a class of the most obstinate and fatal affections arise from, or at least their cause are made more effective by sedentary habits, and habits of inactivity. I allude to scrofulous and tuberculous disease, which I think ought to be classed together. My accomplished friend, Dr. J. N. Graham, at my instance, commenced a series of experiments upon dogs, to see how absolute confinement in a close box would affect them, so that they could not turn around or in any way exercise their limbs, and yet have a good supply of pure air and good food. Although these experiments were not so numerous and varied as to justify positive conclusions, they unquestionably produced the very best specimens of tubercous infiltration in the lungs, as also some of the other organs. These lungs may be seen at his office in Portland Block, by any member of the society who choose to call and examine them. The effect was so complete, and resulted apparently so entirely from confinement, that he is equally with me of the opinion that they stand in the relation of cause and effect. This opinion is strengthened by the fact, that the wild animals caged in the different menageries and zoological gardens die almost invariably from tuberculosis. Observation on the human patients, I think, still fortifies the conclusion. The robust hunter and frontiersman or his family scarcely ever have scrofula or consumption. I have not time and space in this connection to adduce any further evidence of this kind; but I believe reflection on what you have seen, and observation in future, will furnish you with numberless examples of this kind. And this is quite in accordance with physiological laws. Before reminding you of these, however, I will mention a fact with which you all are no doubt familiar: that tuberculous and scrofulous deposits belong to the proteinous compounds, and are identical in chemical composition with these as found in the blood in the shape of albumen, fibrin, etc. Now, the physiological laws to which I have alluded are, 1st, that when an ingredient becomes redundant in the blood, it has a tendency to effusion in the tissues, or completely out of the body; and, 2d, that when an effusion in the tissues takes place, the fluid portions are absorbed, and the solid, becoming compact, act as irritants, and are discharged by suppuration—instance apoplexy, etc. Now, I think, this is just what takes place in tuberculosis; the albumen, which is elaborated in and through the blood, becomes superabundant, because not used by the muscles and nervous system in a state of inactivity, and is effused in the tissues in the shape of tubercles in the lungs and cheesy deposit elsewhere.

Two facts somewhat relevant to this theory is, that in tuberculous persons the proportion of albumen is highly preponderant, and that the chemical composition of muscular fibre is that of albumen. Other causes augment the tuberculous diathesis and determine the location, shape and time of deposit. Irritation from cold would cause an unusual collection of blood in the lungs, and encourage the deposit in them; intestinal irritation would produce the same effect in concentrating the fluid and deposit in the abdomen; premature or morbid excitation in the cranial cavity would induce tuberculous meningitis. May I not also claim the broken-down tuberculous condition, developed by long confinement, from serious accidents, as fractures, crushed limbs, etc., as confirmatory evidence of the theory? But I am prevented by time from discussing this interesting branch of the subject farther. However, I hope, as cultivators of our beloved science, you will examine it with sufficient patience to confirm or falsify the conclusions to which I have been forced by the weight of facts, as I have seen them.

This disturbance of quantitative and qualitative composition of the blood acts in other directions deleteriously. The right composition and relative abundance of separate constituents of the blood are indispensable to the correct physiological action of any of the organs. Hence, we find that indigestion from depraved or insufficient gastric and intestinal secretion, almost invariably attends great inactivity. What digestion does take place is so imperfect as still further to deprave the composition of the fluids, and add to the tendency to organic trouble. The liver, pancreas, and in fact all the glandular apparatus, either become torpid merely or depraved in their action. This indigestion and torpor of the abdominal glands are sometimes alternated with copious effusions in the intestinal canal and diarrhea, as we see in chlorosis and other affections of this sort.

In this general depravation, the nervous system suffers its share. Cephalagia, neuralgia, and neurotic phantasies of all sorts and of the most distressing character, generally accompany this condition of things. The nervous symptoms sometimes assume such prominence as to mask for a time—and I am not sure if they do not postpone other—more disastrous evils to which the patient is tending. Want of exercise has another more direct and positively deleterious influence on the inactive organs. We will be able to appreciate the force and direction of this evil influence best, by recurring to examples of extreme degrees of inactivity. All of us must have observed the great muscular debility with which patients arise from a long confinement, by serious accidents; how long it requires for muscles of a broken limb to regain their wonted vigor, and command of the member, and how much the faculties are all obtunded by the absolute confinement and inactivity of prison life.

It may be stated, I think, without any suspicion of its correctness, that no organ can exist without functional action. They are not developed in animals of corresponding species where they are not needed. The fishes of lightless caverns of America have no eyes, and yet they cannot be proven to be different in any other respect from the fishes in the neighboring external brooks. And I do not hesitate to believe that the sixth generation of human beings confined to an entirely unlighted cavern like the Mammoth Cave, would produce blind offspring. The second having less perfect organs of vision than their parents, the third still greater deteriorations, and so on until the eyes would be merely rudimentary. We have no examples that I am aware, of any of the higher order of animals ever having been submitted to such trial; but I think the inference justifiable, and therefore venture to express it.

The entire inactivity resulting from paralysis is succeeded by complete muscular atrophy—not merely want of energetic nutrition from nervous influence, but structural alteration; the muscles lose their fibrous texture and degenerate into masses of areolar tissue, so that no amount of stimulus is sufficient to cause them to contract. The probability is this, muscular atrophy is one of the greatest obstacles to cure in paralysis.

We cannot account for the cure of muscular paralysis by electricity upon any other principle than its stimulus upon the muscular fibre directly, and their increased capacity therefrom. The more susceptible and powerful the muscular fibre, the less nervous force is necessary to cause contraction. We do not, cannot suppose for a moment, that electricity or galvanism can add to the amount of nervous force, it merely supplies its place temporarily, until the disease which produced the lesion has passed by. Some very valuable practical lessons may be deduced from this consideration of the facts as above stated. The first is that there is no danger of beginning with our electrical stimulation too soon. The stimulation of the muscular fibres by the local application of electricity is necessary (not only useful but necessary) to the preservation of their capacity to act when the nervous stimulus may be restored to them. There is no danger of exhausting their excitability unless we use it imprudently strong. And I insist, that much of the failure resulting from a trial of electricity in paralysis arises from neglect to stimulate the muscular fibres, until their capacity for action is damaged. I am aware that this is not in accordance with the teaching of the majority of authors, as I might show by quotations if I had time, but I hope the members will pardon me for thus plainly stating my convictions.

### THERAPEUTICAL INFLUENCE.

It cannot be doubted that exercise, producing as it does such extensive physiological and pathological effects, may be turned to therapeutic advantage. Its general effects are depurative, alterative and catalytic. I have probably shown sufficiently already the manner in which it is depurative: by promoting secretion and excretion, and thus casting out of the blood, through these emunctories, such effete substances as might be detrimental by their presence. I hope it will not be necessary to dwell on this point to any greater length. It is alterative by being depurative and tonic. It changes the direction and character of morbid action by equalizing the circulation, sending the blood with more vigor through capillaries in which it has been stagnated by congestion, and depleting in a peculiar manner by secretory evacuation. In this last respect we may instance the good effect a hearty commotion of the chest by laughter has, in producing mucous expectoration and relief from constriction, that had previously existed, and the good influence on some diseases of the skin. But still another general effect which I term catalytic, for want of a better term, has a great influence upon the well being of the physical and mental system of man. It is a reacting influence of the different constituents of the blood upon each other, brought about indirectly by exercise. Change in organs produces change in the constitution of the blood, and thus renders it more adapted to the normal nutrition of other organs. If the muscular system remains quiet, the decomposition of certain compounds in the blood containing its nutritive principle does not take place. These compounds may, and probably do contain, in some chemical or vital condition of cohesion or attraction, the principle for the sustenance of some other system of organs, the nervous, I will say, for illustration. But these latter, although present, may not be in a state for appropriation until the former is separated by the muscular nutrition. The play of nutritive affinities between the blood and any of the tissues cannot be perfect unless it be good in all. If there is a failure anywhere in the great circle of nutritive assimilation, constituted by the complete variety which make up the whole system, it is felt by all more or less. Indeed, it is almost certain that the development and perfection of hematosis depend, after the chyle is received into the vessels, upon the influence of all the organs as the blood circulates through their capillaries. In this view of the subject, we see that hematosis is not effected by themes enteric glands, lungs and liver, or any other special set of organs, but is the effect of the co-operation of all the tissues, each contributing to the perfection of the process, by abstracting from and adding to the mass in the general round of circulation. The mesenteric glands, no doubt, impresses upon the chyle some influence in composition and change that is indispensable. Their effect is not only indispensable but peculiar: it is thrown into the blood, mixed and circulated by the heart, passes into the lung to undergo change, which can be effected nowhere else. In the course of its general round it is received into the spleen, where cavernous formation has also a specific effect upon its composition and fitness for use. The liver, with its double circulation, contributes another touch of perfection to the changes already commenced; thence this fluid carries with it a condition not before possessed and acquirable in no other organ, and as has been imagined, perhaps the thymus, thyroid bodies and supra-renal capsules, all contribute in their own way their own impressions, each distinctive and unlike the other; but after all the generally considered processes of sanguification are complete, this blood would be unfit for all its purposes until it had been peculiarly impressed by the cerebral or nervous, the muscular and other tissues. Now what one of these tissues produce a state necessary as precedent circumstance to the changes which succeed in other points, cannot be said with any degree of certainty. But the stomach must not fail in one small particular, in performing its (the) first duties in assimilation; the mesenteric glands, the lungs, the liver, all must act just right; the muscles must act; there must be cerebral, in fact, every variety of functional action, or the blood degenerates in composition and becomes unfit for perfect support to any one of the series of organs. When there is nervous derangement or disease, the blood is deranged in quality and the phosphates are eliminated by the kidneys in more than ordinary quantities, while all the functions are disordered. If the muscles are diseased, as in rheumatism, acid to an inordinate extent may be discovered in the excretions, while the system is disordered in every respect. The kidneys excrete urea under muscular exercise in larger quantities than when in repose. Sedentary and studious habits give preponderance to the phosphates. I have been led into this train of reflection in tracing the mutual influence of one organ upon another through the blood, to exemplify my meaning of catalytic effect (or the decomposing effect) of exercises. I think it shows the necessity of the decomposing effect of one organ to the composing influence of the blood upon others, and that as every cell selects its own peculiar substance and prepares the fluid for another step in development, so does each organ by selecting its own peculiar nutritive properties from the blood, prepare that fluid more completely than it could otherwise be for the sustenance of other organs. Now the general therapeutic effects of exercise in this direction, I think, will be apparent from the above observations. We must enforce muscular exercise when practicable at all, as a conservative measure—a measure to sustain the healthy condition of the well organs. There is no doubt that many serious forms of disease arise from a want of observance of this rule. Many of the derangements referable to the above explanation of facts are called sympathetic derangements. The muscles do not appropriate the substance intended for them, the blood retaining it, and is unfit for the formation of gastric secretion of a perfect character, indigestion results; the secretions from the bowels are slow, costiveness follows, etc. We cannot deny that costiveness and indigestion is the effect of want of exercise, the only difference is the manner of explaining their connection. Want of exercise deranges all the secretions and excretions, they cannot be healthy under a state of absolute confinement, and I think the reason is because the blood is changed, or rather is not sufficiently changed for the perfection of these processes. The special therapeutic effects of exercise are very numerous and varied, and depend upon the kind of exercise, and I will give them more particularly in another place under the head of special exercise.

The kinds of general exercise most in use are carriage, horse and foot. Exercise has also been separated into active and passive, but this is not a strictly correct application of terms, for if there is muscular action at all it is active, and without such action I should not use the term exercise in connection with it. Carriage exercise is a very gentle, or may be made very gentle exercise. The only muscular exercise in carriage riding is the amount of effort necessary to retain position. If sitting and the vehicle is conducted over rough roads and driven pretty rapidly, it becomes very fatiguing on account of the large number of muscles called into play, and the frequency of action necessary to keep position, and may produce a greater general effect, depurative and alterative, than walking. Horseback riding, however, is justly considered the most profitable, because the most varied of any of the three sorts I have mentioned. It calls into action a larger number of muscles simultaneously, perhaps, than any other. The bracing in the stirrups calls into action all the muscles of the lower extremities, those of the back, chest, abdomen, in fact, the whole trunk, including the neck, are employed in maintaining the erect posture, under the varying relations of the centre of gravity; while those of the upper extremity are constantly required to guide, check and otherwise control the animal. Now all the conditions necessary to make this a trying and very fatiguing process is to have a rough gaited and spirited horse. On account of the large number of the muscles employed, and the constant and active efforts required to perform the great diversity of movements, horseback exercise has more general influence than any other with which I am acquainted. It has almost a positive control over deficient secretion and excretion. Indigestion, torpor of the liver and bowels, without organic disease, cannot exist on horseback. It has a very happy effect upon many general affections, by equalizing the circulation, promoting the secretions generally, and energising digestion and nutrition. I know of no one means so subversive of cachectic habits, either with or without organic disease, if it can be borne without immediate detriment, as horseback exercise. An hour a day, ten miles, or any other precise quantum, will often fail, when it will succeed if used to the extent of endurance. If capable, the individual should be on horseback constantly; at any rate, as much as possible. In consequence of the depurative and roborant effects, I look upon it as one of the very best means of preventing and even curing in the early stages of tuberculosis. But in the advanced stages of consumption, it is too fatiguing, the system has lost its capability for enduring so much muscular action, and of course it is not available. It would be foreign to the objects of this paper to go into minute detail of the applicability of this kind of exercise to all the diseases in which it is found to be beneficial. Exercise on foot, or walking, as a therapeutic and hygienic measure, is not as highly appreciated in this country as it is in Europe, particularly in England. The pedestrian feats of many English ladies would, if related, astonish their fair sisters of this country. No doubt much of the robustness of frame and beautiful color of the English females depend upon this salutary habit of walking several miles *every* day. A little wet weather or a bleak wind does not prevent this practice—it would be as inexcusable to neglect the daily walk as the neat toilet exercises which always precede it. Walking is, although more fatiguing than riding on horseback, a less healthful exercise. It is more fatiguing, because some particular muscles support the main burden of the exertion, while others are comparatively at rest. It is less salutary and healthful, because so large an amount of the muscle are not in action as on horseback, and hence the amount of blood change is less;—all the general effects of exercise, however, are to a greater or less extent realized from walking. It is more available than either of the other sorts, because less expensive, and may be practised in less room. Although the influence is much enhanced by open air exercise, yet much valuable results may be brought about in the sick chamber by systematizing this kind of exercise; and in convalescence from acute disease, or in some chronic cases that will not bear exposure, we should not forget it. There is also a kind of exercise too that is available to the poor prostrated bedridden patient that I doubt not may be advantageously used on certain occasions. I allude to what I call static muscular exercise. It consists in the voluntary contraction of the muscular fibres of the limbs, so as to retain them in the position they happen to be placed. The patient with a broken limb, who cannot rise for fear of displacing the adjusted fragments, may thus draw all the fibres of the sound limb tense, and keep them so, or alternately relax and contract them as he may be able to bear it, until all the general effects of muscular exercise be produced, and manifested in the secretions, excretions, appetite, digestion and good health generally. I have no doubt that if patients were rightly instructed and attended to in this respect, many of the broken down constitutions, with which patients often arise from the effects of severe accidents to the extremities, would be prevented. No man can appreciate the powerful effects of this kind of exercise without some experiments upon the subject. I have already alluded to its effects upon the pulse; it also induces perspiration, diuresis, fatigue, hunger, and all the effects of muscular motion. In fact, there is more danger of over exerting the muscles in this way than walking or riding, for in the static exercise of the muscles all of these organs are acting at the same time; in walking, a part of the muscles of the leg carries the foot forward, while those on the posterior part are relaxed and resting; so soon as the foot is planted these muscles contract, and those on the anterior part of the leg relax. And thus they alternately relax and contract, resting half the time. It will be readily experienced that we cannot keep all the muscles of a limb in a state of rigid contraction but a very short time. The larger number of muscles, the greater the general effect. No man can hold all the muscles of the lower extremities in a state of forcible rigidity for two minutes, without blowing and sweating quite as much as if running or walking rapidly. It may seem probably, at least, impracticable to apply this kind of exertion for hygienic or therapeutic purposes, but certainly many patients are so situated that they cannot take any other sort, and their health must suffer. In these cases, I am satisfied, much good can be done in preserving and promoting energy of function in all the more essential organs by this static mode of affecting them. Besides these sorts of what may be called general exercise, because not necessarily confined to any set of muscles, there is what may be called special exercise, having reference to some part of the system; thus, by exercising the muscles of the arms and chest, it is expected that the lungs will be expanded beyond their former size; in exercising the abdominal, we may promote the action of the alimentary canal and quicken digestion; by dancing, we can enlarge and develop the muscles of the hips and legs; by fencing and boxing, the arms, and so on. Much may no doubt be effected in this way, which may be made useful under certain circumstances. Gymnastics, as practised by skilful teachers, is designed to be of general and special usefulness.

Although every studied motion and act has a special reference to some part of the body or system of organs, they are so numerous and so skilfully designed, as to have a developing effect upon the muscular system as a whole, by operating upon each part successively, while they have the depurating, excretory, alterative and catalytic effects that are produced by general exercise. All young persons, who are too indolent and proud to work, and all others whose occupation is deficient in exercise, should avail themselves of this, as an excellent means of promoting their health. Occupations often involve the exercise of only a part of the muscular systems, and hence may be used under certain circumstances, therapeutically, for changing the current of circulation and strengthening parts of the organism; so that we should never lose sight of the excellent effect of a change of employment that may be brought about under certain contingencies. And I cannot but think that exercise by some employment, with the double object of pecuniary and hygienic good, is more effective when properly adapted to the case, than merely to take exercise for our health.

I cannot do more than merely allude to this view of the subject now on account of the limit of this paper. I may say in a general way in conclusion, that exercise in acute diseases, particularly inflammations, is scarcely ever applicable, absolute rest being the rule, and that it is to chronic cases to which it is most appropriately adapted. Acute cases, in fact, are nearly all injured by the arterial excitement which accompanies it, but after the acute symptoms to some extent have subsided, exercise will promote healthy action and assist in repairing the damage resulting from the force of the attack, and should be prescribed with as much care and discrimination as the more medicinal part of treatment. This will often be found as essential to the welfare of our patient as any other remedial measure. Many cachexia, no doubt, grow out of the anemic and deranged condition of the system resulting from the progress of acute diseases, and might be prevented by this and other roborant means during convalescence. Great care, however, will be necessary to avoid relapse from too much arterial excitement; the return to exercise must be gradual, and cautiously watched. There is probably no chronic disease in which exercise may not be employed as a remedy, and I think it is the principal curative means in many of them. On the lungs, exercise has a peculiarly good influence. They of course partake in the general catalytic operations, while they grow and enlarge from the increased demands made upon them for oxygenation of a greater amount of blood.

